# Supplementary material for: Enhancing NSCLC recurrence prediction with PET/CT habitat imaging, ctDNA, and integrative radiogenomics-blood insights
Source: Nat Commun. 2024 Apr 11;15:3152. doi: 10.1038/s41467-024-47512-0 (PMC11009351; doi:10.1038/s41467-024-47512-0)
Supplement: Supplementary file 1 — Supplementary Information [file 41467_2024_47512_MOESM1_ESM.pdf]

Supplementary Information

Enhancing NSCLC Recurrence Prediction with PET/CT Habitat Imaging, ctDNA, and Integrative Radiogenomics-blood Insights

Sheeba J. Sujit[†], Muhammad Aminu[†], Tatiana V. Karpinets, Pingjun Chen, Maliazurina B. Saad, Morteza Salehjahromi, John D. Boom, Mohamed Qayati, James M. George, Haley Allen, Mara B. Antonoff, Lingzhi Hong, Xin Hu, Simon Heeke, Hai T. Tran, Xiuning Le, Yasir Y. Elamin, Mehmet Altan, Natalie I. Vokes, Ajay Sheshadri, Julie Lin, Jianhua Zhang, Yang Lu, Carmen Behrens, Myrna C.B. Godoy, Carol C. Wu, Joe Y. Chang, Caroline Chung, David A. Jaffray, Ignacio I. Wistuba, J. Jack Lee, Ara A. Vaporciyan, Don L. Gibbons, John Heymach, Jianjun Zhang [‡], Tina Cascone[‡], Jia Wu[‡, ✉]

† These authors contributed equally: Sheeba J. Sujit, Muhammad Aminu
‡ These authors jointly supervised this work: Jianjun Zhang, Tina Cascone, Jia Wu

Contents

Supplementary Figures 1-9

Supplementary Tables 1-10

Supplementary Figures

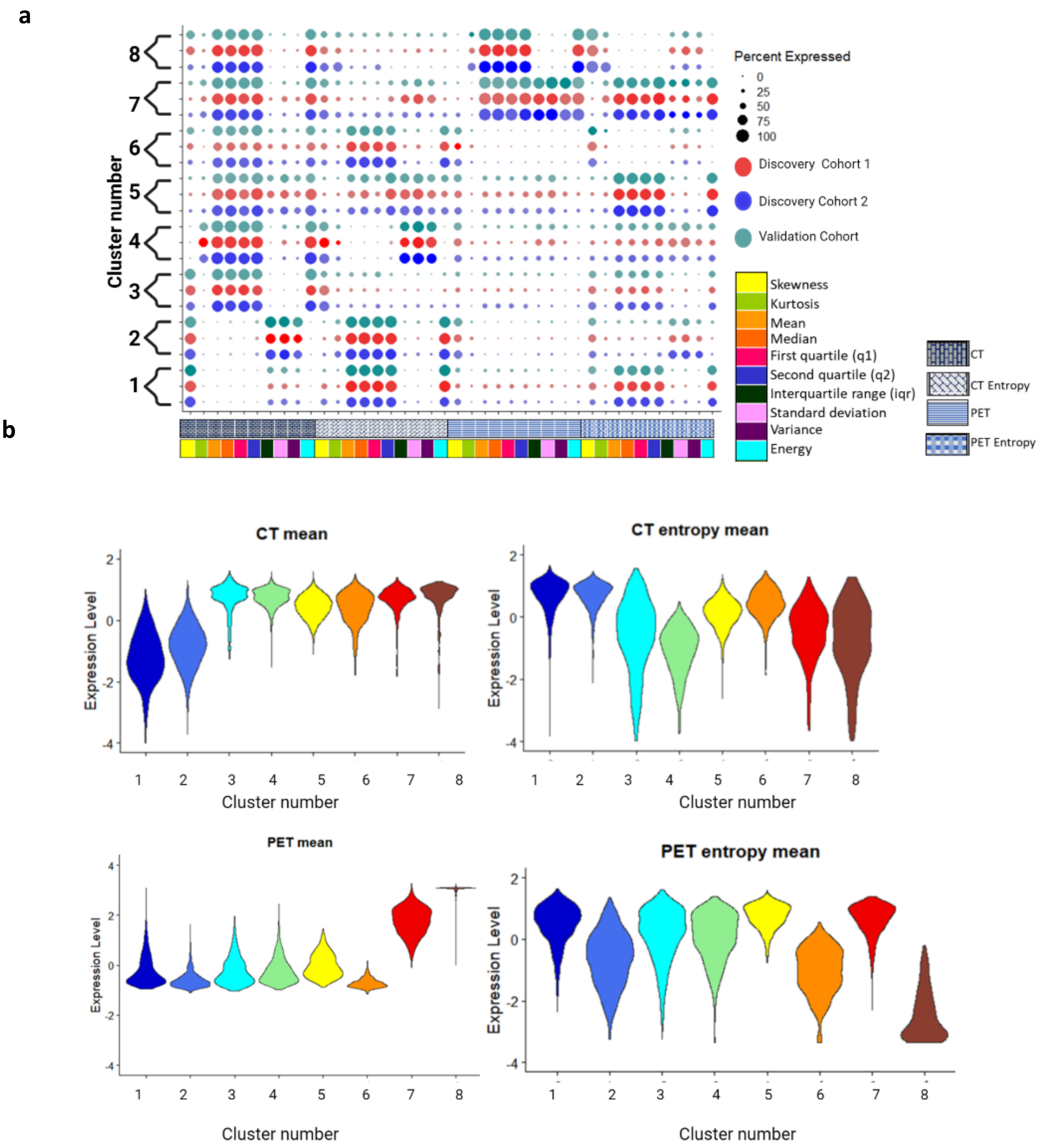

Supplementary Fig. 1. Feature vectors across different tumor subregions (clusters)

a. Dot plot showing correlation of the 40-dimensional feature vectors with individual clusters in each cohort. **b.** Violin plots showing the relationship of mean values of CT, CT Entropy, PET and PET Entropy across different clusters. Source data are provided in Supplementary Data 7.

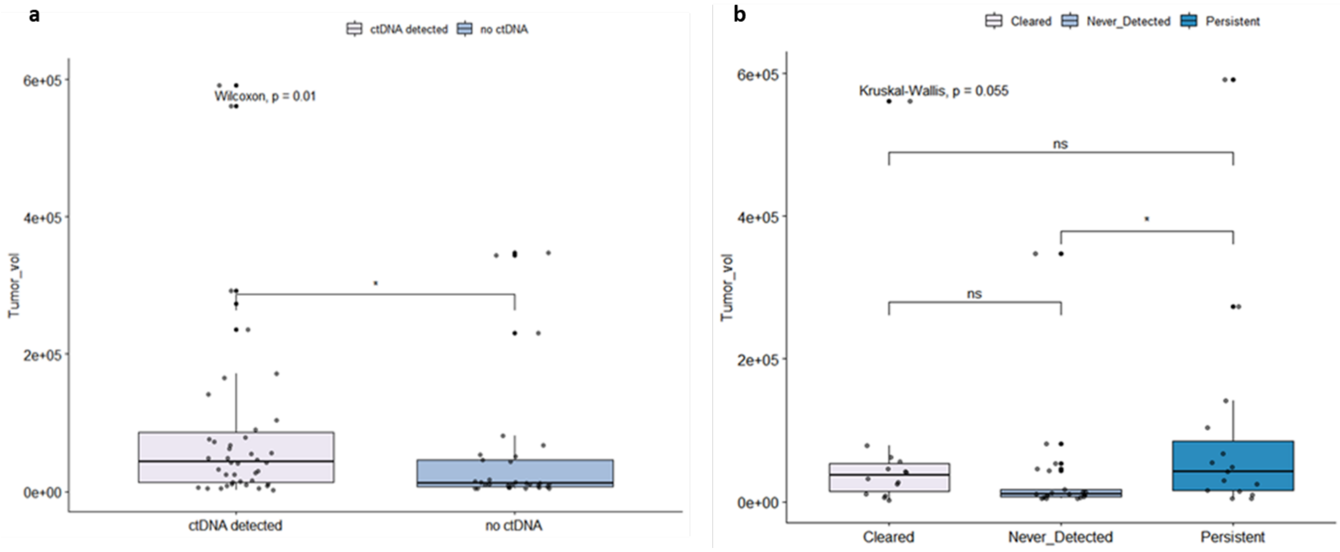

Supplementary Fig. 2. Correlations of tumor volume with the ctDNA data

a. Significant difference in baseline tumor volume when stratified by baseline ctDNA profiling status (*P* = 0.01) was observed. **b.** No significant difference was observed in baseline tumor volume when stratified by serial ctDNA clearance status (*P* = 0.055). Source data are provided in Supplementary Data 8.

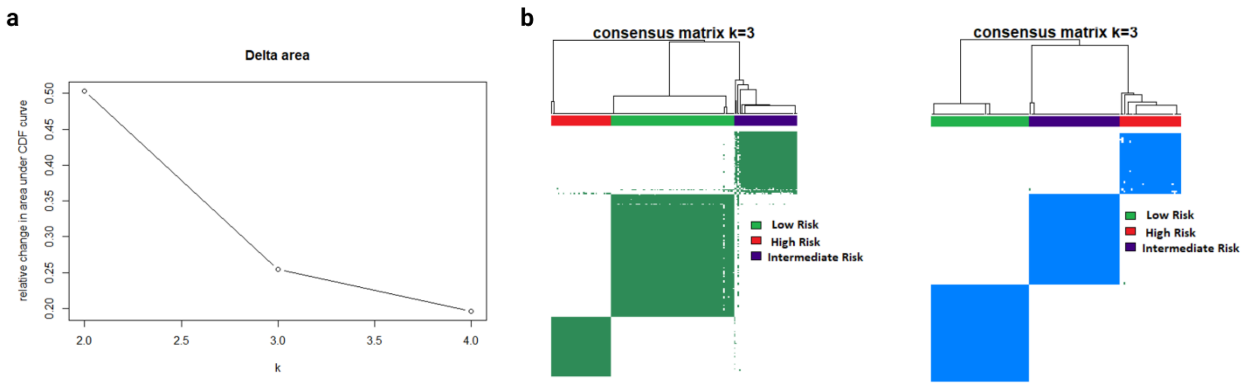

Supplementary Fig. 3. Consensus clustering identifies three habitat imaging subtypes in discovery and validation cohorts

a. All patients were classified using consensus matrix = 3. The optimal solution (k=3) provided the smallest incremental change in the area under the cumulative distribution function (CDF) curve while maximizing consensus within subtypes **b.** Consensus matrix heat map depicting consensus values on a white-to-green color scale of each cluster on the discovery cohort. Consensus matrix heat map depicting consensus values on a white-to-blue color scale of each cluster on the validation cohort. Source data are provided in Supplementary Data 9.

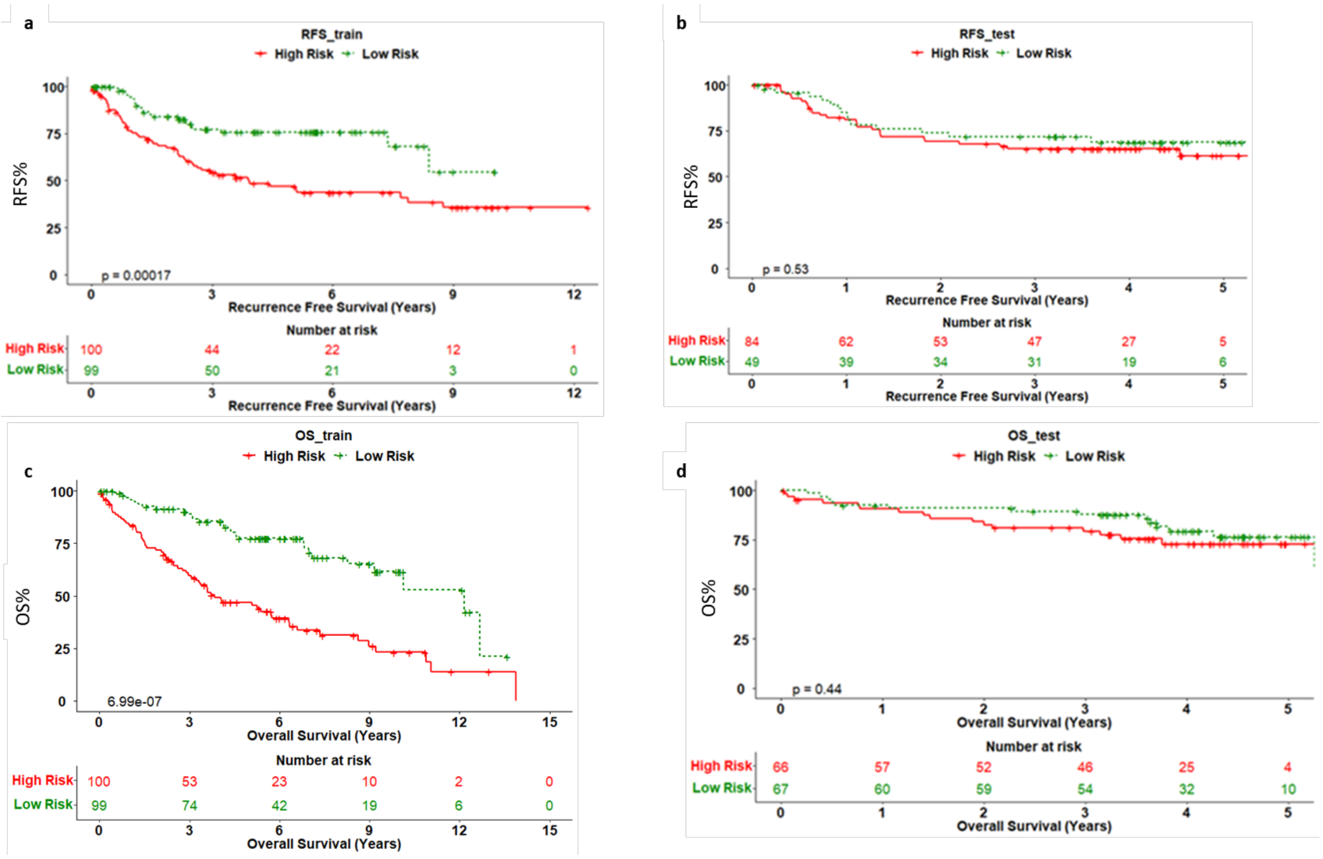

Supplementary Fig. 4. Comparison of habitat-based tumor subtype prediction with classical radiomics approach

Kaplan-Meier curves for recurrence-free survival using classical radiomic features in discovery **(a)** and internal validation **(b)** cohorts are shown. The classical radiomics model did not achieve robust stratification during validation (P = 0.53). Kaplan-Meier curves for overall survival using classical radiomic features in discovery **(c)** and internal validation **(d)** cohorts also shows poor performance of the model to stratify patients on the validation cohort (P = 0.44). Source data are provided in Supplementary Data 10.

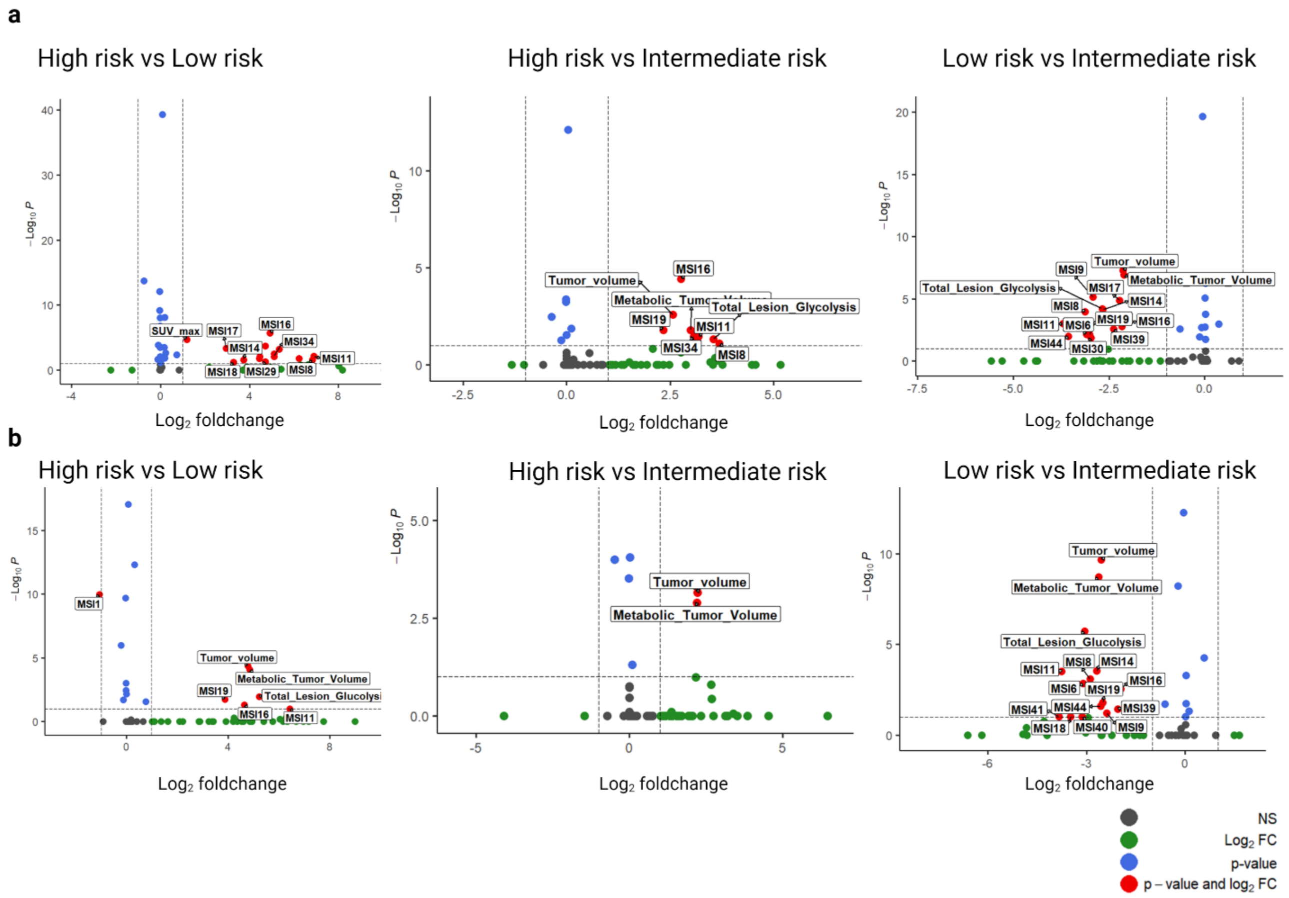

Supplementary Fig. 5. MSI features are associated with stratifying different risk groups in NSCLC

a. Volcano plots are shown for all three pairwise comparisons of risk groups in the discovery cohort with the numbers of expressed features. Features in red indicate significant differential expression based on adjusted *P* values of <0.05, and a log2 foldchange of >±0.25. The top MSI and conventional imaging features are labeled. **b.** Volcano plots are shown for all three pairwise comparisons of risk groups in the validation cohort with the numbers of expressed features. No MSI feature was prominent in the high- versus intermediate-risk pair. Source data are provided in Supplementary Data 11.

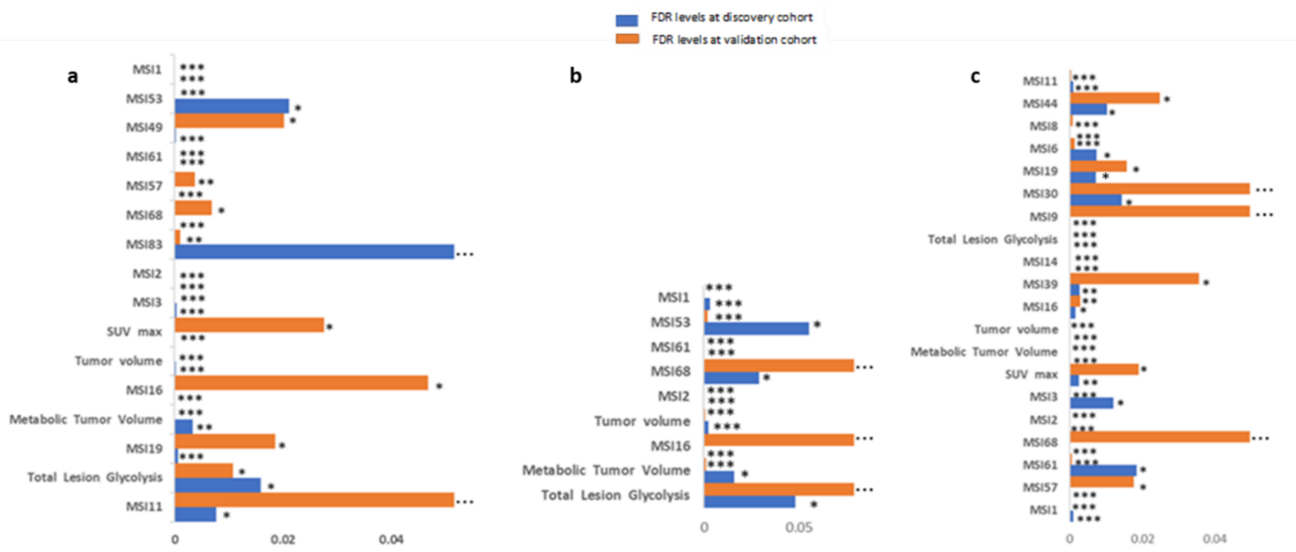

Supplementary Fig. 6. Bar plot shows false discovery rate (FDR) levels of key imaging features compared between different risk groups

Bar plot showing key features compared between high- and low-risk groups (**a**), high- and intermediate-risk groups (**b**), and low- and intermediate-risk groups (**c**). * *P* < 0.05; ** *P* < 0.005, *** *P* < 0.001; ··· *P* > 0.05. Source data are provided in Supplementary Data 12.

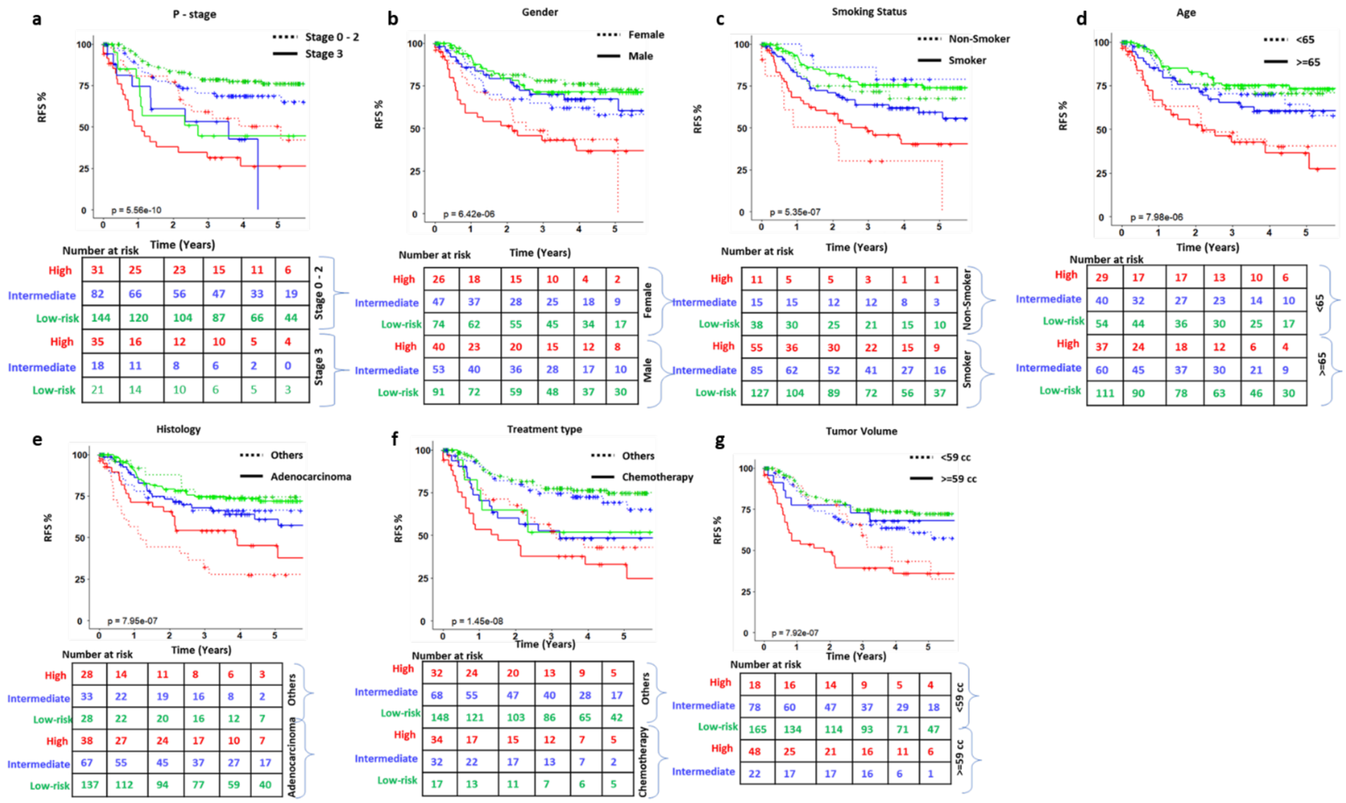

Supplementary Fig. 7. RFS prediction performance of the habitat imaging model in subgroups of clinicopathological features in the integrated cohort.

Kaplan-Meier curves show prognostic prediction performance in subgroups of (a) p-stage (b) gender (c) smoking status (d) age (e) histology and (f) treatment type (g) tumor volume. Source data are provided in Supplementary Data 13.

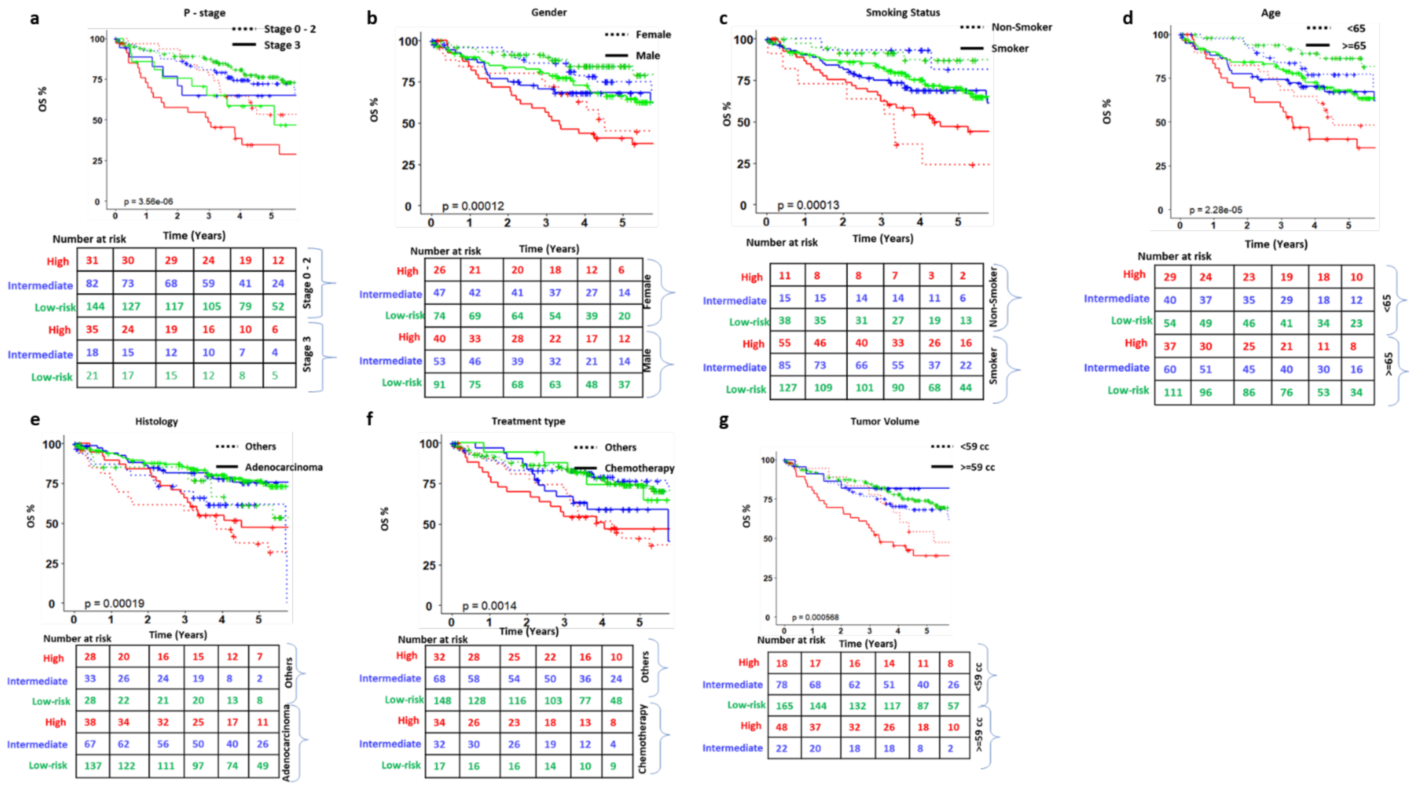

Supplementary Fig. 8. OS prediction performance of the habitat imaging model in subgroups of clinicopathological features in the integrated cohort.

Kaplan-Meier curves show prognostic prediction performance in subgroups of (a) p-stage (b) gender (c) smoking status (d) age (e) histology and (f) treatment type (g) tumor volume. Source data are provided in Supplementary Data 13.

.

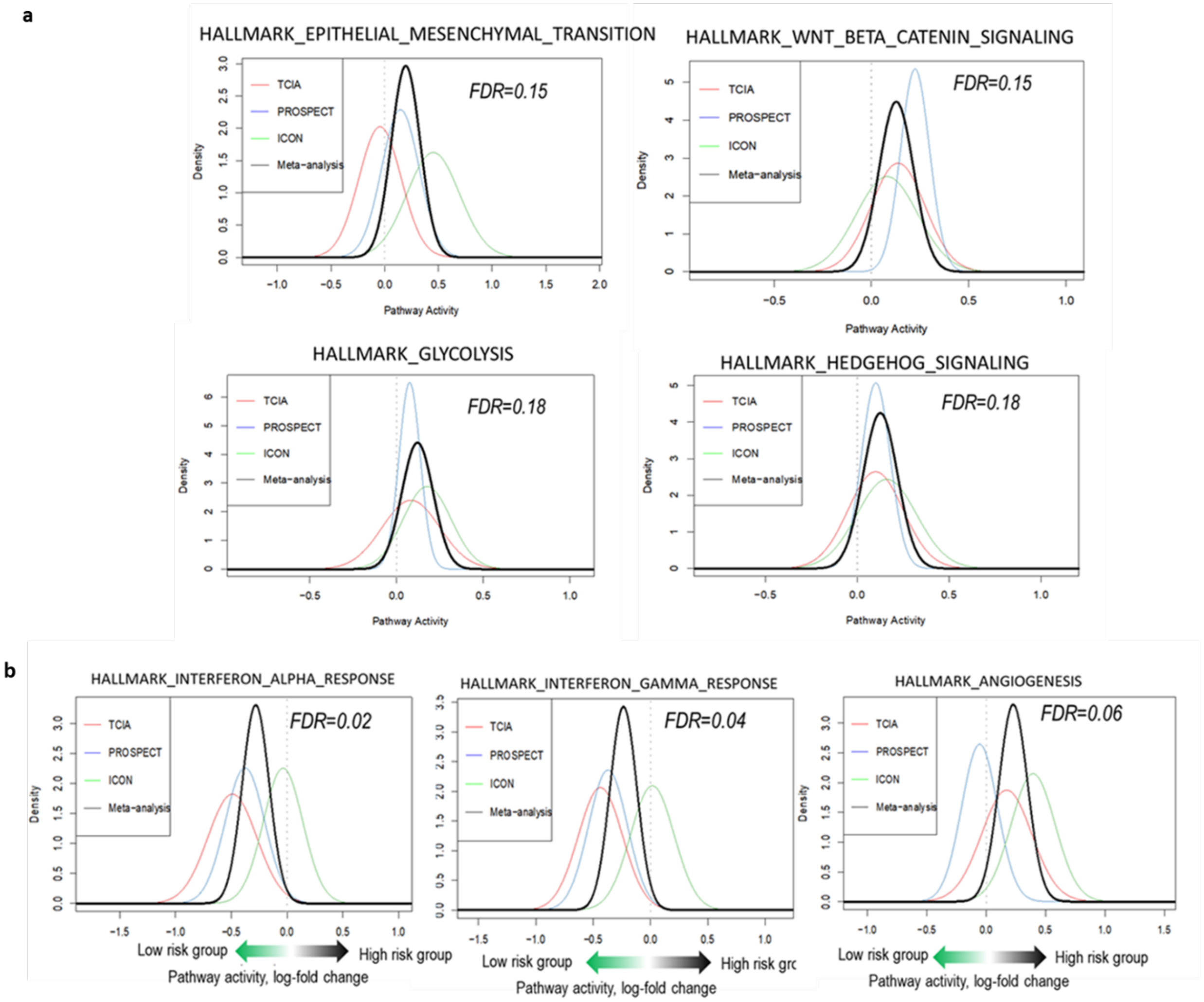

Supplementary Fig. 9. Gene set enrichment analysis in the integrated cohort

a. Enriched genes in the high-risk versus low-risk groups. The high-risk group showed significant enrichment of HALLMARK_EPITHELIAL_MESENCHYMAL_TRANSITION (FDR = 0.15), and two signaling pathways, Wnt/β-catenin and Hedgehog signaling, which are known to regulate EMT and well-known pathways associated with NSCLC progression. No hallmark pathways were significantly enriched in comparisons of the intermediate-risk versus high-risk groups or the intermediate-risk versus low-risk groups.

b. Density plots of 3 enriched pathways in the integrated cohort: HALLMARK_INTERFERON_ALPHA_RESPONSE (FDR = 0.02), HALLMARK_INTERFERON_GAMMA_RESPONSE (FDR = 0.04), and HALLMARK_ANGIOGENESIS (FDR = 0.06).

Supplementary Tables

Supplementary Table 1. The OS and RFS net reclassification improvement (NRI) metric shows that radiomics using habitat imaging perform significantly better (0.216 (P = 0.04); 0.257 (P = 0.03)). Source data are provided in Supplementary Data 1.

**Imaging model**	**NRI (OS)**	**NRI (RFS)**
Classical radiomics approach	Reference	
Habitat Imaging approach	0.216 (P = 0.04)	0.257 (P = 0.03)

Supplementary Table 2. Univariate and multivariate Cox regression analysis of recurrence-free survival (RFS) and overall survival (OS) in the discovery cohort. Multivariate analysis included adjustment for tumor volume. Low risk subtype was prognostic of RFS (HR = 0.35, *P* = 0.000904 ***). All statistical tests were 2-sided, with *P* < 0.05 indicative of a statistically significant difference. Source data are provided in Supplementary Data 2.

**Univariate and multivariate analyses of RFS in discovery cohort**				
Variable	RFS			
	Univariate		Multivariate	
	P value	HR (95% CI)	P value	HR (95% CI)
High – Risk (n=46)	Reference			
Intermediate – Risk (n=52)	0.01*	0.47 (0.26 – 0.84)	0.059	0.54 (0.28 – 1.02)
Low -Risk (n=101)	9.69e-06 ***	0.30 (0.17 – 0.51)	0.000904 ***	0.35 (0.19 – 0.65)
Tumor volume (n=199)	0.000185 ***	1.3 (1.1 – 1.5)	0.295	1.10 (0.92 – 1.31)
**Univariate and multivariate analyses of OS in discovery cohort**				
Variable	OS			
	Univariate		Multivariate	
	P value	HR (95% CI)	P value	HR (95% CI)
High – Risk (n=46)	Reference			
Intermediate – Risk (n=52)	0.151	0.68 (0.40 – 1.15)	0.374	0.77 (0.43 – 1.4)
Low -Risk (n=101)	0.014*	0.54 (0.33 – 0.88)	0.104	0.62 (0.35 – 1.1)
Tumor volume (n=199)	0.021*	1.2 (1 – 1.4)	0.331	1.09 (0.91 – 1.3)

Supplementary Table 3. Univariate and multivariate Cox regression analysis of recurrence-free survival (RFS) and overall survival (OS) in the validation cohort. Multivariate analysis included adjustment for tumor volume. Low risk subtype was prognostic of RFS and OS (HR = 0.15, *P* = 0.011**; HR = 0.11, *P* = 0.005**). All statistical tests were 2-sided, with *P* < 0.05 indicative of a statistically significant difference. Source data are provided in Supplementary Data 2.

**Univariate and multivariate analyses of RFS in validation cohort**				
Variable	RFS			
	Univariate		Multivariate	
	P value	HR (95% CI)	P value	HR (95% CI)
High – Risk (n=18)	Reference			
Intermediate – Risk (n=50)	0.085	0.49 (0.22 – 1.10)	0.036*	0.26 (0.072 – 0.91)
Low -Risk (n=64)	0.007**	0.33 (0.14 – 0.74)	0.011**	0.15 (0.033 – 0.64)
Tumor volume (n=132)	0.167	1.2 (0.94 – 1.4)	0.247	0.75 (0.461 – 1.22)
**Univariate and multivariate analyses of OS in validation cohort**				
Variable	OS			
	Univariate		Multivariate	
	P value	HR (95% CI)	P value	HR (95% CI)
High – Risk (n=18)	Reference			
Intermediate – Risk (n=50)	0.036*	0.4 (0.17 – 0.94)	0.03*	0.24 (0.065 – 0.87)
Low -Risk (n=64)	0.00098 ***	0.2 (0.08 – 0.53)	0.005**	0.11(0.023 – 0.51)
Tumor volume (n=132)	0.037*	1.2 (1 – 1.5)	0.344	0.80 (0.504 – 1.27)

Supplementary Table 4. The correlation of simple tumor-related parameters such as MTV, TLG, and tumor volume with the habitat imaging subtypes. 58%, 59%, and 64% correlation was observed between habitat subtypes and stratification by MTV, TLG or tumor volume, respectively; while a sizable of patients are off-diagonal. These results suggest habitat analysis learns novel patterns beyond these conventional CT and PET metrics. Source data are provided in Supplementary Data 3.

**MTV vs Habitat**			
	MTV_High	MTV_Intermediate	MTV_Low
High risk subtype	80%	16%	5%
Intermediate risk subtype	57%	34%	9%
Low risk subtype	2%	39%	59%
**TLG vs Habitat**			
	TLG_High	TLG_Intermediate	TLG_Low
High risk subtype	80%	13%	8%
Intermediate risk subtype	51%	39%	10%
Low risk subtype	4%	38%	58%
**Tumor Volume vs Habitat**			
	TV_High	TV_Intermediate	TV_Low
High risk subtype	86%	14%	0
Intermediate risk subtype	54%	42%	4%
Low risk subtype	0	36%	64%

Supplementary Table 5. Univariate and multivariate Cox regression analysis of recurrence-free survival (RFS) in the integrated cohort. Multivariate analysis included adjustment for tumor and lobe location. High-risk and intermediate-risk imaging subtypes were prognostic of RFS (HR = 0.43, *P* = 0.000906 ***; HR = 0.30, *P* = 4.37e-06 ***) in the multivariate analysis. Only the right upper lobe is prognostic in our cohorts, which is independent of habitat imaging. All statistical tests were 2-sided, with *P* < 0.05 indicative of a statistically significant difference. Source data are provided in Supplementary Data 4.

**Univariate and multivariate analyses of RFS integrated cohort**				
Variable	RFS			
	Univariate		Multivariate	
	P value	HR (95% CI)	P value	HR (95% CI)
**Imaging Subtypes**				
Low – Risk (n = 62)	Reference			
Intermediate – Risk (n = 164)	8.49e-08***	0.30 (0.19 – 0.46)	4.37e-06 ***	0.30 (0.18 – 0.50)
High –Risk (n = 92)	0.000507***	0.43 (0.27 – 0.69)	0.000906 ***	0.43 (0.26 – 0.71)
**Tumor location**				
Central (n = 70)	Reference			
Peripheral (n = 202)	0.131	0.71 (0.45 – 1.1)	0.637	0.89 (0.55 – 1.44)
Ultracentral (n = 46)	0.583	1.18 (0.66 – 2.1)	0.263	0.71 (0.39 – 1.29)
**Lung side/Lobe location**				
Left lower lobe (n = 50)	Reference			
Left upper lobe (n = 79)	0.907	0.97 (0.55 – 1.70)	0.663	0.88 (0.49 – 1.57)
Right lower lobe (n = 51)	0.494	0.80 (0.42 – 1.51)	0.239	0.68 (0.36 – 1.29)
Right middle lobe (n = 22)	0.122	1.74 (0.86 – 3.49)	0.27	1.50 (0.73 – 3.06)
Right upper lobe (n = 116)	0.028*	0.52 (0.30 – 0.93)	0.032*	0.53 (0.30 – 0.95)

Supplementary Table 6. Univariate and multivariate Cox regression analysis of overall survival (OS) in the integrated cohort. Multivariate analysis included adjustment for tumor and lobe location. High-risk and intermediate-risk imaging subtypes were prognostic of OS (HR = 0.45, *P* = 0.001***; HR = 0.35, *P* = 5.74e-05***). Only the right upper lobe is prognostic in our cohorts, which is independent of habitat imaging. All statistical tests were 2-sided, with *P* < 0.05 indicative of a statistically significant difference. Source data are provided in Supplementary Data 4.

**Univariate and multivariate analyses of OS integrated cohort**				
Variable	OS			
	Univariate		Multivariate	
	P value	HR (95% CI)	P value	HR (95% CI)
**Imaging Subtypes**				
Low – Risk (n = 62)	Reference			
Intermediate – Risk (n = 164)	2.29e-05***	0.39 (0.25 – 0.60)	5.74e-05***	0.35 (0.21 – 0.59)
High –Risk (n = 92)	0.003**	0.49 (0.31 – 0.78)	0.001**	0.45 (0.27 – 0.73)
**Tumor location**				
Central (n = 70)	Reference			
Peripheral (n = 202)	0.296	0.79 (0.51 – 1.2)	0.861	0.96 (0.60 – 1.54)
Ultracentral (n = 46)	0.932	0.97 (0.53 – 1.8)	0.109	0.60 (0.32 – 1.12)
**Lung side/Lobe location**				
Left lower lobe (n = 50)	Reference			
Left upper lobe (n = 79)	0.445	0.81 (0.47 – 1.39)	0.337	0.76 (0.44 – 1.33)
Right lower lobe (n = 51)	0.294	0.72 (0.39 – 1.33)	0.161	0.64 (0.35 – 1.19)
Right middle lobe (n = 22)	0.692	1.15 (0.57 – 2.32)	0.996	1.00 (0.48 – 2.06)
Right upper lobe (n = 116)	0.012*	0.50 (0.29 – 0.86)	0.015*	0.51 (0.29 – 0.88)

Supplementary Table 7. Univariate and multivariate Cox regression analysis of recurrence-free survival (RFS) in the discovery cohort. Multivariate analysis included adjustment for radiotherapy-related parameters. Intermediate-risk imaging subtypes was prognostic of RFS (HR = 0.36, *P* = 0.001**) in the multivariate analysis. No correlation was observed between radiotherapy - related parameters and survival after adjusting them in multivariate analysis. All statistical tests were 2-sided, with *P* < 0.05 indicative of a statistically significant difference. Source data are provided in Supplementary Data 5.

**Univariate and multivariate analyses of RFS in discovery cohort**				
Variable	RFS			
	Univariate		Multivariate	
	P value	HR (95% CI)	P value	HR (95% CI)
**Imaging Subtypes**				
Low – Risk (n = 40)	Reference			
Intermediate – Risk (n = 47)	0.004**	0.40 (0.21 – 0.74)	0.001**	0.36 (0.189 – 0.67)
High –Risk (n = 58)	0.069	0.61 (0.35 – 1.04)	0.051	0.58 (0.334 – 1.00)
**Post-operative Radiotherapy (POR)**				
Patients did not receive POR (n = 118)	Reference			
Patients received POR (n = 27)	0.102	1.6 (0.91 – 2.8)	0.499	0.50 (0.069 – 3.67)
**Radiotherapy dosage**				
No dosage (n = 118)	Reference			
≤ 54 Gy (n = 17)	0.18	1.6 (0.81 – 3.1)	0.266	3.23 (0.410 – 25.48)
> 54 Gy (n = 7)	0.046*	2.5 (1.01 – 6.4)	0.097	6.24 (0.717 – 54.27)

Supplementary Table 8. Univariate and multivariate Cox regression analysis of overall survival (OS) in the discovery cohort. Multivariate analysis included adjustment for radiotherapy-related parameters. High and Intermediate – risk subtypes remained prognostic of OS (HR = 0.47, *P* = 0.009**; HR = 0.28, *P* = 0.000378***). No correlation was observed between radiotherapy - related parameters and survival after adjusting them in multivariate analysis. All statistical tests were 2-sided, with *P* < 0.05 indicative of a statistically significant difference. Source data are provided in Supplementary Data 5.

**Univariate and multivariate analyses of OS in discovery cohort**				
Variable	OS			
	Univariate		Multivariate	
	P value	HR (95% CI)	P value	HR (95% CI)
**Imaging Subtypes**				
Low – Risk (n = 40)	Reference			
Intermediate – Risk (n = 47)	0.001**	0.31 (0.15 – 0.63)	0.000378***	0.28 (0.1396 – 0.57)
High –Risk (n = 58)	0.042*	0.57 (0.34 – 0.98)	0.009**	0.47 (0.2714 – 0.83)
**Post-operative Radiotherapy (POR)**				
Patients did not receive POR (n = 118)	Reference			
Patients received POR (n = 27)	0.284	1.4 (0.75 – 2.7)	0.495	0.30 (0.009 – 9.36)
**Radiotherapy dosage**				
No dosage (n = 118)	Reference			
≤ 54 Gy (n = 17)	0.519	1.3 (0.58 – 2.9)	0.449	3.86 (0.116 – 127.66)
> 54 Gy (n = 7)	0.051	2.5 (1.00 – 6.4)	0.11	17.58 (0.52 – 589.48)

Supplementary Table 9. Univariate and multivariate Cox regression analysis of recurrence-free survival (RFS) in the discovery cohort. Multivariate analysis included adjustment for EGFR mutation and ALK fusion. Intermediate-risk imaging subtypes was prognostic of RFS (HR = 0.35, P = 0.001**) in the multivariate analysis. The oncogene mutation status exhibits no correlation with patient survival. All statistical tests were 2-sided, with $P < 0.05$ indicative of a statistically significant difference. Source data are provided in Supplementary Data 6.

**Univariate and multivariate analyses of RFS in discovery cohort**				
Variable	RFS			
	Univariate		Multivariate	
	P value	HR (95% CI)	P value	HR (95% CI)
**Imaging Subtypes**				
Low – Risk (n = 40)	Reference			
Intermediate – Risk (n = 47)	0.004**	0.40 (0.21 – 0.74)	0.001**	0.35 (0.18 – 0.67)
High –Risk (n = 58)	0.069	0.61 (0.35 – 1.04)	0.057	0.59 (0.35 – 1.02)
**EGFR mutation**				
Wild Type (n = 120)	Reference			
Mutant (n = 25)	0.275	1.4 (0.77 – 2.5)	0.097	1.66 (0.91 – 3.03)
**Anaplastic Lymphoma Kinase (ALK)**				
Wild Type (n = 144)	Reference			
Fusion (n = 1)	0.63	1.6 (0.23 – 12)	0.283	3.05 (0.40 – 23.41)

Supplementary Table 10. Univariate and multivariate Cox regression analysis of overall survival (OS) in the discovery cohort. Multivariate analysis included adjustment for EGFR mutation and ALK fusion. Intermediate-risk imaging subtypes was prognostic of OS (HR = 0.32, *P* = 0.001**) in the multivariate analysis. The oncogene mutation status exhibits no correlation with patient survival. All statistical tests were 2-sided, with *P* < 0.05 indicative of a statistically significant difference. Source data are provided in Supplementary Data 6.

**Univariate and multivariate analyses of RFS and OS in discovery cohort**				
Variable	OS			
	Univariate		Multivariate	
	P value	HR (95% CI)	P value	HR (95% CI)
**Imaging Subtypes**				
Low – Risk (n = 40)	Reference			
Intermediate – Risk (n = 47)	0.001**	0.31 (0.15 – 0.63)	0.001**	0.32 (0.162 – 0.65)
High –Risk (n = 58)	0.042*	0.57 (0.34 – 0.98)	0.013*	0.50 (0.287 – 0.86)
**EGFR mutation**				
Wild Type (n = 120)	Reference			
Mutant (n = 25)	0.45	0.75 (0.36 – 1.6)	0.81	0.91 (0.42 – 1.94)
**Anaplastic Lymphoma Kinase (ALK)**				
Wild Type (n = 144)	Reference			
Fusion (n = 1)	0.688	0.36 (0.0026 – 50)	0.831	0.57 (0.0031 – 104.05)
