# Supplementary material for: Enhancing NSCLC recurrence prediction with PET/CT habitat imaging, ctDNA, and integrative radiogenomics-blood insights
Source: Nat Commun. 2024 Apr 11;15:3152. doi: 10.1038/s41467-024-47512-0 (PMC11009351; doi:10.1038/s41467-024-47512-0)
Supplement: Supplementary file 3 — Reporting Summary [file 41467_2024_47512_MOESM3_ESM.pdf]

nature portfolio

Corresponding author(s): Jia Wu

Last updated by author(s): Feb 6, 2024

Reporting Summary

Nature Portfolio wishes to improve the reproducibility of the work that we publish. This form provides structure for consistency and transparency in reporting. For further information on Nature Portfolio policies, see our Editorial Policies and the Editorial Policy Checklist.

Statistics

For all statistical analyses, confirm that the following items are present in the figure legend, table legend, main text, or Methods section.

n/a	Confirmed	
☐	☒	The exact sample size (*n*) for each experimental group/condition, given as a discrete number and unit of measurement
☐	☒	A statement on whether measurements were taken from distinct samples or whether the same sample was measured repeatedly
☐	☒	The statistical test(s) used AND whether they are one- or two-sided<br>*Only common tests should be described solely by name; describe more complex techniques in the Methods section.*
☐	☒	A description of all covariates tested
☐	☒	A description of any assumptions or corrections, such as tests of normality and adjustment for multiple comparisons
☐	☒	A full description of the statistical parameters including central tendency (e.g. means) or other basic estimates (e.g. regression coefficient) AND variation (e.g. standard deviation) or associated estimates of uncertainty (e.g. confidence intervals)
☐	☒	For null hypothesis testing, the test statistic (e.g. *F*, *t*, *r*) with confidence intervals, effect sizes, degrees of freedom and *P* value noted<br>*Give P values as exact values whenever suitable.*
☒	☐	For Bayesian analysis, information on the choice of priors and Markov chain Monte Carlo settings
☐	☒	For hierarchical and complex designs, identification of the appropriate level for tests and full reporting of outcomes
☐	☒	Estimates of effect sizes (e.g. Cohen's *d*, Pearson's *r*), indicating how they were calculated

Our web collection on statistics for biologists contains articles on many of the points above.

Software and code

Policy information about availability of computer code

Data collection	PET/CT data are collected from The Cancer Imaging Archive or PACS at MD Anderson. Patient clinical and follow-up information are collected from The Cancer Imaging Archive or EPIC at MD Anderson.
Data analysis	We used MATLAB (2022b) and python (3.10.5) for image processing,R software (version 4.1.2) for the statistical and correlative analysis. Custom codes will be publicly released at Zenodo https://doi.org/10.5281/zenodo.10611536 The Habitat Imaging code is available at https://github.com/WuLabMDA/Habitat-Analysis

For manuscripts utilizing custom algorithms or software that are central to the research but not yet described in published literature, software must be made available to editors and reviewers. We strongly encourage code deposition in a community repository (e.g. GitHub). See the Nature Portfolio guidelines for submitting code & software for further information.

Data

Policy information about availability of data

All manuscripts must include a data availability statement. This statement should provide the following information, where applicable:

- Accession codes, unique identifiers, or web links for publicly available datasets
- A description of any restrictions on data availability
- For clinical datasets or third party data, please ensure that the statement adheres to our policy

Source data are provided with this paper. The source data for Figs. 3,4 6, Tables. 1,3,4, Supplementary Figs. 1-8 and Supplementary Tables 1-5 are provided as a Source Data file. The FDG-PET/CT and clinical data of TCIA cohort are publicly available on The Cancer Imaging Archive https://wiki.cancerimagingarchive.net/

display/Public/NSCLC+Radiogenomics#28672347d6e83195f69f438ca0d1a3d20fbc450d. The raw FDG-PET/CT and clinical data of ACRIN 6668/RTOG 0235 cohort are publicly available on The Cancer Imaging Archive https://wiki.cancerimagingarchive.net/pages/viewpage.action?pageId=39879162#398791626e061ab3228446d59c8ce2ac2d1aa117 . The raw FDG-PET/CT of ICON and PROSPECT are not publicly shared to protect patient privacy, but are available for research use from the corresponding author. MTA is required to be approved by MD Anderson committees by providing the research plan and is restricted to non-commercial academic research purposes. Request can be submitted to J.W. and will receive an internal review response within 30 days. In addition, anonymized data and the input for the predictive models are available at Zenodo https://doi.org/10.5281/zenodo.1061153659. Deidentified ctDNA data for patients in the internal validation cohort are available in Source data for Fig 6. The genomics data of PROSPECT cohort are available at GEO repository GSE42127 https://www.ncbi.nlm.nih.gov/geo/query/acc.cgi?acc=GSE42127. The genomics data of TCIA cohort are available at GEO repository GSE103584 https://www.ncbi.nlm.nih.gov/geo/query/acc.cgi?acc=GSE103584. The genomics data of ICON cohort are available at [UPLOADING IT NOW]. The remaining data are available within the Article, Source Data, Supplementary Information, and Supplementary Data files. Source data are provided with this paper.

Research involving human participants, their data, or biological material

Policy information about studies with human participants or human data. See also policy information about sex, gender (identity/presentation), and sexual orientation and race, ethnicity and racism.

Reporting on sex and gender	This retrospective study is a gender-based analysis. Gender information was collected from medical record and used as a main variable for multivariate adjustment and subgroup analysis.
Reporting on race, ethnicity, or other socially relevant groupings	The retrospective study contains race and ethnicity information, which are used as main factor in our multivariate adjustment.
Population characteristics	The details of population are presented in Table 1
Recruitment	The patients were obtained from in-house MD Anderson databased as well as public datasets
Ethics oversight	MD Anderson IRB approval was obtained

Note that full information on the approval of the study protocol must also be provided in the manuscript.

Field-specific reporting

Please select the one below that is the best fit for your research. If you are not sure, read the appropriate sections before making your selection.

[x] Life sciences [ ] Behavioural & social sciences [ ] Ecological, evolutionary & environmental sciences

For a reference copy of the document with all sections, see nature.com/documents/nr-reporting-summary-flat.pdf

Life sciences study design

All studies must disclose on these points even when the disclosure is negative.

Sample size	no sample size calculation. We applied strict patient inclusion criteria as shown in Figure 1. The robust performance of habitat imaging subtypes were demonstrated in discovery and validation sets (Figure 4) as well as univariate and multivariate analyses (Table 3 and Table 4)
Data exclusions	the detailed exclusions for each cohort were presented in Figure 1. We excluded patients without PET/CT scans, stage IV disease, without follow up information.
Replication	we have four independent cohorts (figure 1). The subtypes were identified in discovery cohorts, and further validated in MD Anderson testing set as well as the ACRIN trial set.
Randomization	This is a multicenter retrospective study without randomization. Two cohorts were used for discovery purpose, and two independent cohorts for validation (Figure 1).
Blinding	This is a retrospective study. During model development and validation, the clinical outcome data were blinded to assess the clinical values of habitat imaging subtypes

Reporting for specific materials, systems and methods

We require information from authors about some types of materials, experimental systems and methods used in many studies. Here, indicate whether each material, system or method listed is relevant to your study. If you are not sure if a list item applies to your research, read the appropriate section before selecting a response.

Materials & experimental systems

n/a	Involved in the study
☒	☐ Antibodies
☒	☐ Eukaryotic cell lines
☒	☐ Palaeontology and archaeology
☒	☐ Animals and other organisms
☒	☐ Clinical data
☒	☐ Dual use research of concern
☒	☐ Plants

Methods

n/a	Involved in the study
☒	☐ ChIP-seq
☒	☐ Flow cytometry
☒	☐ MRI-based neuroimaging

Plants

Seed stocks	*Report on the source of all seed stocks or other plant material used. If applicable, state the seed stock centre and catalogue number. If plant specimens were collected from the field, describe the collection location, date and sampling procedures.*
Novel plant genotypes	*Describe the methods by which all novel plant genotypes were produced. This includes those generated by transgenic approaches, gene editing, chemical/radiation-based mutagenesis and hybridization. For transgenic lines, describe the transformation method, the number of independent lines analyzed and the generation upon which experiments were performed. For gene-edited lines, describe the editor used, the endogenous sequence targeted for editing, the targeting guide RNA sequence (if applicable) and how the editor was applied.*
Authentication	*Describe any authentication procedures for each seed stock used or novel genotype generated. Describe any experiments used to assess the effect of a mutation and, where applicable, how potential secondary effects (e.g. second site T-DNA insertions, mosiacism, off-target gene editing) were examined.*
