# Supplementary material for: Enhancing NSCLC recurrence prediction with PET/CT habitat imaging, ctDNA, and integrative radiogenomics-blood insights
Source: Nat Commun. 2024 Apr 11;15:3152. doi: 10.1038/s41467-024-47512-0 (PMC11009351; doi:10.1038/s41467-024-47512-0)
Supplement: Supplementary file 4 — Supplementary Data Description [file 41467_2024_47512_MOESM4_ESM.docx]

**Supplementary Data 1**

Description:

Integrated Cohort Data for OS and RFS net reclassification improvement (NRI) metric calculation

**Supplementary Data 2**

Description:

Discovery and Validation Cohort Data for Univariate and multivariate Cox regression analysis of recurrence-free survival (RFS) and overall survival (OS)

**Supplementary Data 3**

Description:

Tumor-related parameters such as MTV, TLG and tumor volume of patients from integrated cohort arranged in ascending order of their range.

**Supplementary Data 4**

Description:

Integrated cohort data for Univariate and multivariate Cox regression analysis of recurrence-free survival (RFS) and (OS). Multivariate analysis included adjustment for tumor and lobe location.

**Supplementary Data 5**

Description:

Discovery cohort data for Univariate and multivariate Cox regression analysis of recurrence-free survival (RFS) and overall survival (OS). Multivariate analysis included adjustment for radiotherapy-related parameters.

**Supplementary Data 6**

Description:

Discovery cohort data for Univariate and multivariate Cox regression analysis of recurrence-free survival (RFS) and overall survival (OS). Multivariate analysis included adjustment for EGFR mutation and ALK fusion.

**Supplementary Data 7**

Description:

Mean values of CT, CT Entropy, PET and PET Entropy across different clusters.

**Supplementary Data 8**

Description:

Discovery cohort data of baseline tumor volume with baseline ctDNA profiling status and ctDNA clearance status.

**Supplementary Data 9**

Description:

MSI features to construct the consensus matrix heat map extracted from discovery cohorts TCIA, PROSPECT and validation cohort ICON.

**Supplementary Data 10**

Description:

Discovery and Validation cohorts’ data for recurrence-free survival and overall survival analysis using classical radiomics approach.

**Supplementary Data 11**

Description:

Discovery and Validation cohorts’ features for pairwise comparison of risk groups

**Supplementary Data 12**

Description:

False discovery rate (FDR) levels of key imaging features compared between different risk groups.

**Supplementary Data 13**

Description:

Integrated cohort data for RFS and OS prediction performance of the habitat imaging model in subgroups
